# Communication Experiences of Patients With Hearing Loss During Hemodialysis Treatment and the Potential Role of Communication Tools: A Qualitative Study

**DOI:** 10.1177/20543581261454470

**Published:** 2026-06-12

**Authors:** Alex DeBusschere, Meaghan Lunney, Sonja Reid, Nancy Verdin, Shannan Love, Gillian Crysdale, Stephanie Thompson, David Nicholas, Tiffany Boulton, Kara Schick-Makaroff, Lorienne Jenstad, Sharon Straus, Jayna Holroyd-Leduc, Maoliosa Donald, Patti-Jo Sullivan, Tanis Howarth, Julie Evans, Marcello Tonelli

**Affiliations:** 1 Department of Medicine, University of Calgary, AB, Canada; 2 Patient Partner, Calgary, AB, Canada; 3 Faculty of Rehabilitation Medicine, University of Alberta, Edmonton, Canada; 4 Department of Medicine, University of Alberta, Edmonton, Canada; 5 Faculty of Social Work, University of Calgary, AB, Canada; 6 Department of Community Health Sciences, University of Calgary, AB, Canada; 7 Faculty of Nursing, University of Alberta, Edmonton, Canada; 8 School of Audiology and Speech Sciences, The University of British Columbia, Vancouver, Canada; 9 Department of Medicine, University of Toronto, ON, Canada; 10 Alberta Health Services, Edmonton, Canada

**Keywords:** hemodialysis, hearing loss, patient-provider communication, qualitative research, communication support, hémodialyse, perte auditive, communication patient-prestataire, recherche qualitative, aide à la communication

## Abstract

**Background:**

People with hearing loss may have difficulty communicating with health care providers if not properly supported. Hearing loss is common among people with kidney failure. Outpatient hemodialysis centers may present communication barriers due to noisy machines and overlapping conversations. Tools, such as assistive listening devices, exist to help people with hearing loss communicate. If and how they should be used in the outpatient hemodialysis setting is unclear. Understanding the patient perspective is an important first step before implementing such solutions.

**Objective:**

Describe the communication-related experiences of patients with hearing loss when conversing with health care providers during hemodialysis treatment, focusing on perceptions about communication tools.

**Design:**

Qualitative descriptive.

**Setting:**

Outpatient hemodialysis centers in Calgary and Edmonton, Alberta, Canada.

**Participants:**

Adults with kidney failure receiving maintenance hemodialysis with self-reported hearing loss.

**Methods:**

Semi-structured individual interviews. Interviews were audio-recorded, transcribed, and abductively coded using a validated communication framework, a strategy to guide communication access in practice, and participants’ experiences.

**Results:**

Fourteen patients participated between October 2023 and January 2024. Patient perceptions about communication tools varied. We identified three themes that describe these differences: (1) communication tools may be needed in transitional or clinically complex situations, (2) patients with their own resources may rely less on center-provided tools, and (3) awareness and self-advocacy for support varies across patients.

**Limitations:**

The major limitation of this study is the lack of representation from patients with language barriers and those belonging to the Deaf community or with overlooked hearing difficulties. Consequently, results may not be transferable to all patients with hearing loss in Alberta or elsewhere.

**Conclusions:**

Communication support needs are both person-specific and context-dependent, varying across and within patients. Not all patients that may benefit from communication tools will be comfortable asking or accepting help. Clinicians should routinely check in with patients about their communication needs and offer a variety of tools to accommodate as needed.

## Abrégé

**Contexte:**

La perte auditive, fréquente chez les personnes atteintes d’insuffisance rénale, peut compliquer la communication avec les prestataires de soins en l’absence d’un soutien adapté. Les unités d’hémodialyse ambulatoires constituent des environnements propices aux difficultés de communication en raison du bruit des machines et du chevauchement des conversations. Des outils d’aide à la communication, comme les aides de suppléance à l’audition, peuvent contribuer à améliorer la communication. L’indication et les modalités d’utilisation de ces outils en hémodialyse ambulatoire demeurent mal définies. La prise en compte de la perspective des patients constitue une étape fondamentale avant leur déploiement.

**Objectifs:**

Décrire les expériences de communication des patients présentant une perte auditive pendant les traitements d’hémodialyse, en mettant l’accent sur leurs perceptions des outils d’aide à la communication.

**Conception:**

Étude qualitative descriptive.

**Cadre:**

Centres d’hémodialyse ambulatoire à Calgary et Edmonton, en Alberta (Canada)

**Personnes participantes:**

Des adultes atteints d’insuffisance rénale et traités par hémodialyse, présentant une déficience auditive autodéclarée.

**Méthodologie:**

Les entretiens individuels semi-structurés ont été enregistrés, transcrits et analysés par codage abductif à l’aide d’un cadre de communication validé, d’une stratégie guidant l’accès à la communication en pratique et des expériences rapportées par les participants

**Résultats:**

Quatorze patients ont participé à l’étude entre octobre 2023 et janvier 2024. Les perceptions des patients à l’égard des outils d’aide à la communication variaient. Nous avons dégagé trois éléments permettant de décrire ces différences : (1) des outils d’aide à la communication peuvent s’avérer nécessaires dans les situations de transition ou présentant une complexité clinique, (2) les patients ayant leurs propres ressources dépendent moins des outils fournis par le centre, et (3) le degré de sensibilisation aux outils disponibles et la capacité à demander du soutien varient d’un patient à l’autre.

**Limites:**

La principale limite de cette étude est le manque de représentation des patients présentant des barrières linguistiques, ainsi que de ceux appartenant à la communauté sourde ou ayant des troubles auditifs non reconnus. Par conséquent, les résultats peuvent ne pas être transférables à tous les patients atteints de perte auditive en Alberta ou ailleurs.

**Conclusion:**

Les besoins en matière d’aides à la communication dépendent à la fois de chaque individu et du contexte, et varient d’un patient à l’autre, voire chez une même personne. Tous les patients susceptibles d’en bénéficier ne seront pas nécessairement à l’aise pour demander ou accepter de l’aide. Les cliniciens devraient évaluer régulièrement les besoins des patients et offrir divers outils pour y répondre.

## Introduction

Good communication is a critical component of health care. People with hearing loss often experience difficulties conversing with health care providers,^1-3^ resulting in more medical errors and complications,^4,5^ emergency visits, hospitalizations, and readmissions,^6,7^ worse physical and mental health outcomes,^8^ and decreased patient satisfaction.^9^

People with kidney disease are at high risk of developing hearing loss. An estimated 45.3% of patients receiving outpatient maintenance hemodialysis have some degree of hearing loss.^10^ Furthermore, these centers present additional barriers to communication such as an open-room layout, noisy machines, and overlapping conversations.

Communication tools, such as assistive listening devices or captioning, help people with hearing loss communicate. However, whether and how they can support patients during outpatient hemodialysis treatment is unknown. In this study, we describe the communication-related experiences of patients with hearing loss when conversing with health care providers during hemodialysis treatment, focusing on perceptions about communication tools.

## Methods

### Study Design

This qualitative descriptive study^11,12^ used individual semi-structured interviews and is reported according to Consolidated Criteria for Reporting Qualitative Research (COREQ) guidelines^13^ (Supplemental Table S1).

### Research Team and Reflexivity

The study team was comprised of health services and nephrology researchers and staff, clinicians, patient partners, and provincial leaders in Communication Access. The core team involved in data collection and analysis included five members. AD is a female research coordinator with a MSc in behavioral neuroscience. ML is a female research associate with a PhD in health services research. GC is a BSc student research trainee. SR is a female patient partner with experience of hearing loss and cochlear implant use. NV is a female patient partner with a BSc in Occupational Therapy (BScOT) and a certificate in Patient and Community Engagement Research (PACER). She lives with hearing loss, wears hearing aids, and receives maintenance hemodialysis at a center in Alberta. All were trained in qualitative research and have been involved in prior work exploring how health services can better support patients with hearing loss.

### Participants and Recruitment

We purposively recruited^14^ people with hearing loss that were receiving maintenance hemodialysis from one of six centers in Calgary or Edmonton, Alberta. Nursing staff first screened patients and identified a selection of potentially eligible patients based on predefined criteria: 18 years of age or older, sufficient cognitive capacity to provide informed consent, a current Alberta address, and suspected hearing loss (based on the nurse’s observations). Patients belonging to the Deaf community (i.e., individuals who participate in the culture, society, and language of Deaf people, which is based on Sign language^15^) were excluded. A research coordinator (AD) then approached eligible patients to introduce herself, describe the goals of the study and what participation would entail, and confirm self-reported hearing loss. Patients were informed that if they chose to participate, they would have the option of an in-person (at the dialysis center) or online (at the location of their choice) interview and for both, a Communication Access Realtime Translation (CART) professional was available if requested. AD conducted the interviews. No patients had an established relationship with AD prior to the study.

AD reviewed the characteristics of the sample throughout recruitment and continued to conduct interviews until we achieved a representative sample based on self-reported age, gender, hemodialysis vintage, socioeconomic status, comorbidities, and hearing device use. Despite offering translation services, no eligible patients with language barriers agreed to participate. AD recruited patients until thematic saturation^16^ was achieved by analyzing interview data throughout recruitment. We continued to interview additional patients until we felt no new concepts were emerging, as described below.

The University of Calgary Conjoint Health Research Ethics Board (REB22-1818) approved this study, and all participants provided written informed consent.

### Data Collection and Analysis

Before beginning the interview, the research coordinator gave each participant a paper demographics form with questions about their self-reported sociodemographics, comorbidities, hearing device use, and number of years on hemodialysis. An open-ended question captured additional comments. No field notes were collected.

Members of the research team (AD, ML, SR, VR) designed the interview guide (Supplemental Material, Item 1) to align with the objectives of the study. A research coordinator (AD) conducted and audio-recorded the interviews. Each participant was interviewed once over a 30- to 60-minute session. All interviews were completed bedside while patients were receiving hemodialysis. While all efforts were made to keep conversations private, non-participants such as other patients, health care providers, or non-consenting support persons were occasionally present while interviews were being conducted. Comments from non-participants who were present were not included in our analyses. A professional transcriptionist transcribed the audio files verbatim. Transcripts were not returned to participants for correction.

We used an abductive thematic approach^17^ to analyze the data so that themes were identified using a combination of existing literature (deductive) and participant experiences (inductive). Data were first deductively coded based on (1) the Feldman-Stewart Framework for Communication,^18^ a validated conceptual framework that describes the factors that influence patient-provider communication, and (2) the Alberta Health Services 6 Strategies for Communication Access resource,^19^ which provides health care workers suggestions on how to care for patients with communication challenges, including hearing loss. We then inductively added new codes based on participants’ experiences. Codes were grouped and consolidated into broader themes.

Three coders (AD, GC, NV) independently coded de-identified transcripts using Microsoft Word or NVivo 14. Coders met regularly to compare and discuss emerging findings and resolve any discrepancies. Coding occurred iteratively until consensus was reached. After initial coding was completed, members of the research team (AD, ML, MD, SR, NV) met to discuss codes, resolve discrepancies, identify patterns, and produce a final list of themes.

### Methodological Rigor

We took several actions to establish the credibility, transferability, dependability, and confirmability^20^ of this work. Interviews were audio-recorded and transcribed verbatim. Multiple coders were involved and findings were triangulated to ensure interpretations matched respondent views. Although member-checking was not done, patient partners were involved in the coding and consensus discussions to formulate the themes and interpretations. We clearly describe the specific context in which this study took place and recruitment limitations so that readers can be informed about how the findings may apply elsewhere. Recruitment was audited and coders developed a codebook to document how codes were defined and applied. NVivo was used to document how transcripts were coded and how themes were formed from the codes.

## Results

Forty-five patients were invited to participate in the study. Sixteen consented to participate. One requested to withdraw from the study due to difficulty hearing the interviewer despite use of an assistive listening device and a second was excluded after the interview as it was later realized they did not meet eligibility criteria (identified as Deaf, not as having hearing loss). A total of 14 patients participated between October 2023 and January 2024 (Table 1, Supplemental Table S2).

**Table 1.** Participant Characteristics (N = 14).

Characteristic	Participants, no. (%)
**Age, median [IQR], y**	76 [73-81]
**Gender**	
Man	9 (64)
Woman	5 (36)
**Community**	
Urban/suburban	12 (86)
Rural/remote	2 (14)
**Living situation**	
Lives alone	4 (29)
Household with others	6 (43)
Residential facility	3 (21)
Continuing care	1 (7)
**Hemodialysis vintage** ^a^	
< 1 year	4 (29)
1-3 years	4 (29)
4-6 years	2 (14)
>6 years	4 (29)
**Hearing devices worn**	
Yes, hearing aid	9 (64)
No hearing device	5 (36)
**Additional disabilities**	
Vision	7 (50)
Communication	4 (29)
Reading	5 (36)
Mobility	7 (50)
Memory/understanding	4 (29)
**Difficulty with technology**	
Yes	6 (43)
No	8 (57)
**Language barriers**	
Yes	0 (0)
No	14 (100)
**Socioeconomics**	
Difficulty paying for basic needs	
Yes	4 (29%)
No	10 (71%)
Health care coverage	
Insufficient	7 (50%)
Sufficient	6 (43%)
Prefer not to answer	1 (7%)

a Number of years a person has been receiving hemodialysis.

Overall, participants were satisfied with communication at the hemodialysis center. “It’s been pretty good. I cannot really say that I don’t hear [providers] or that I don’t get across what I sort of want” (Participant 2). “I can communicate with whoever I needed to . . . I can communicate with the nurses quite well” (Participant 4). Many felt that simply asking providers to repeat or come closer was sufficient. “I just ask them to come closer” (Participant 6).When asked if communication tools would be helpful on site, perspectives were mixed. Many expressed doubt. “I don’t think [tools are] necessary. It’s easy for [providers] to come closer to me, so I can answer them” (Participant 6). “I’m really good at lip reading now” (Participant 4). Others felt they would be helpful. “One of the nurses at the [centre] actually brought a pencil and paper [to] write stuff down for me . . . it was nice to see that they made an effort” (Participant 1). “It’s noisy. If it wasn’t for that [pocketalker], I would be in trouble” (Participant 12).

**Figure 1.**
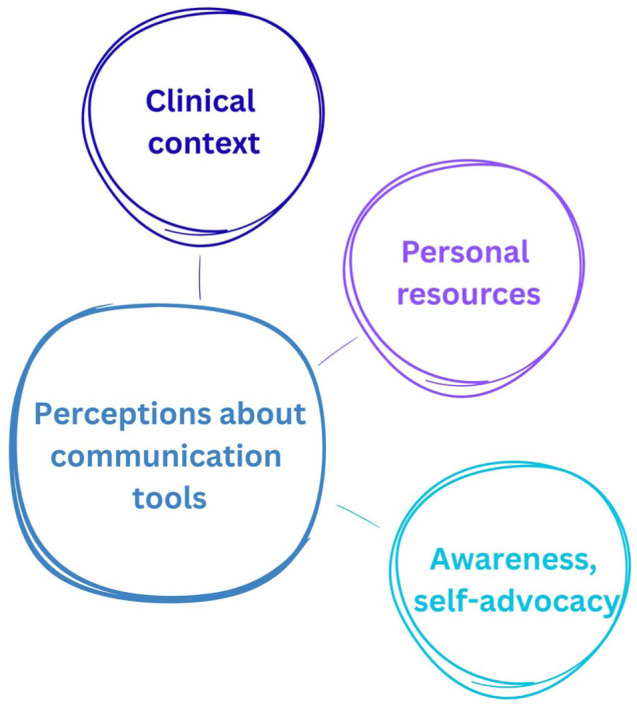
Factors shaping patient communication experiences during hemodialysis and the potential for communication tools. *Note*. **Clinical context: Theme 1.** Communication tools may be needed more during transitional or clinically complex situations. **Personal resources: Theme 2.** Patients with their own resources may rely less on center-provided tools. **Awareness, self-advocacy: Theme 3.** Awareness and self-advocacy for support varies across patients.

We identified 3 themes that describe patient perceptions about communication tools during hemodialysis (Figure 1): (1) communication tools may be needed in transitional or clinically complex situations, (2) patients with their own resources may rely less on center-provided tools, and (3) awareness and self-advocacy for support varies across patients. Table 2 presents additional participant quotations to support each theme.

**Table 2.** Additional Participant Quotations for Each Theme.

Theme 1: communication tools may be needed in transitional or clinically complex situations
“Originally at first, yes. [I had difficulty understanding]. Yeah, it would have been nice to have [help]. Because when I first started dialysis there wasn’t really any signs, they pointed, they did that. But, if you can’t really find out what’s going on then it’s really tough, or hard, not tough, but hard to get in.” (Participant 1)
“They are going to reduce one of my medications. So, that’s a good thing if I can hear that because otherwise, I just won’t get it right . . . If you don’t [hear] then you might get the wrong medication . . . then the blood work will probably go off the charts and I will be in danger of doing harm to myself.” (Participant 3)
“Awhile back one of the doctors changed my blood pressure med[ication] . . . I continued to take that med[ication] the old way and then when I came in, I asked one of the nurses . . . She went through the information and . . . clarified with me and I said, ok, I was under the impression either [the nephrologist] made a mistake or the pharmacy made a mistake . . . I thought, oh, ok, this med[ication change] is right, but you know, I did question it because I just kind wasn’t sure . . . [the nephrologist] never took time to explain that to me.” (Participant 2)
“There is horror stories about that in any medical setting that it was misheard . . . typical example is, I get a shot of iron, they reminded me of that today, that I [don’t] have to do anything . . . there have been instances that patients have, they have a supply of iron at home, but because they are doing it here means you should [not] be doing it at home. So, you have to be aware of what you are being told all the time . . . writing down changes to medications [would be helpful].” (Participant 3)
“When I was originally losing [my hearing], I brought a pad of paper, or I would usually have a pad of paper and pencil in my pocket so I could write back and forth if that day I wasn’t hearing very well.” (Participant 1)
“Sometimes it’s important information that I’m missing . . . I’ll miss the appointment because I didn’t really hear quite what they said or I’ll misread what they said.” (Participant 7)
“I can communicate with the nurses quite well. There’s not much dialogue between us except they ask the same things for their information you know . . . they have to go through a little questionnaire every time you sit down here. ‘Are you bleeding from anywhere? Have you had any hormonal change? Are your lungs still working? Are your ankles swollen? Are you feeling dizzy?’ They have the same questions all the time and I think if you didn’t answer them or you answer it the wrong way, I think they would know right off the bat.” (Participant 4)
Theme 2: patients with their own resources may rely less on center-provided tools
“When I didn’t have hearing aids [communication tools] would have helped a lot . . . [Now] I’m pretty set with the hearing aids. It’s not really anything that can help.” (Participant 1)
“If I didn’t have such good hearing aids, I may not be as happy. I’ve got high quality hearing aids so I don’t have any problems.” (Participant 14)
“I got hearing aids and [communication] was much, much better.” (Participant 5)
“I don’t think it’s a major impediment for me as long as I have [my hearing aids] with me . . . as long as these hearing aids continue to work, I think I’ll be ok . . . a lot of my hearing loss has been taken care of just with these.” (Participant 13)
“I can’t afford to buy new [hearing aids]. I would like a [assistive listening device]. It would be ok when I’m here . . . you can hear people.” (Participant 10)
“If I could do something with my ears, pay thousands of dollars for hearing aids, I’d do that. But I can’t, so I deal with it.” (Participant 8)
“A hearing aid . . . how much is it going to cost? $2000 – 3000? Forget it . . . No way in the word I can afford [hearing aids]. If I can get something like this [assistive listening device], it’s no problem . . . I can turn up the volume and no problem . . . It’s a godsend. I can hear everything, it’s perfect. That’s all I need.” (Participant 12)
“It’s harder if there is a lot of ambient noise, but it’s not as bad as when I had the less technologically advanced devices. I can hear better with these [new] ones than with the old ones.” (Participant 13)
“[Hearing aids] are expensive, they are really expensive and if you are financially strapped, there is no coverage for it. They give you 1000 bucks or something. Fortunately, I got these at no cost.” (Participant 13)
“I had hearing aids before and then I lost one, but my AISH only pays for them every five years. They worked really well. I think [the solution] is to get my new hearing aids. I bought a voice amplifier one time and it worked, but it wasn’t really great quality. It distorted a lot of things.” (Participant 7)
“If I had to buy [new hearing aids], I would really have to stop and think about whether I could do that.” (Participant 14)
“I don’t wear them here for the simple fact is that I fall asleep and they whistle sometimes . . . I’ll say to [the nurses] – I don’t have my hearing aids in today, so then they’ll speak up a little bit more.” (Participant 5)
“I should be wearing my hearing aids, but I don’t wear them here because they bother me too much and there’s no problem . . . If it’s something I’m not understanding . . . I just look at my wife and she can hear very well and I have no problem communicating . . . I’ve been fortunate because my wife is always here and if I look at her, she tells me what they are asking and then there’s no problem.” (Participant 4)
“My wife, she’ll answer the question for the nurse . . . I tell them and my wife will answer the questions. So then we have no difficulty and they don’t ask me, they will ask my wife instead of asking me . . . She is with me every day, every time we are in here, so she knows what is going on.” (Participant 6)
Theme 3: awareness and self-advocacy for support varies across patients
“I didn’t think [letting my healthcare providers know about my hearing loss] would matter at first. And then when I found out I wasn’t knowing what was really going on, I let them know.” (Participant 1)
“If I really need to know something, I guess, I get them to repeat. I’ll just tell them ‘I’m sorry, I don’t hear well. Can you repeat that?’” (Participant 10)
“I think a lot of it has to do with the person themselves to say . . . ‘I didn’t hear you, can you repeat that? Or, you know, I’m at a loss today. Could you talk louder?’ . . . I think a lot of it falls, like I say, on the patient. If I don’t make you aware of whatever is going on with me, then how do you know what to do?” (Participant 2)
“Sometimes I get shy, you know? Like the one time when all the students came. Sometimes I don’t want to just jump up and talk.” (Participant 11)
“You have to tell [healthcare providers] because they don’t know my problem . . . they don’t know until I tell them.” (Participant 12)
“I would tell them ‘I’m deaf, I can barely hear and so you have to speak slow and annunciate clearly and get in close, and speak right into the right side.” (Participant 13)
“I’m not afraid to say ‘Well, I can’t hear what you’re saying. So repeat it.’ . . . if I can’t hear their voices, I’ll tell them to speak up. I’m not afraid, I’m not shy. If you don’t speak up, you don’t get looked after . . . I can see a person without, you know, that’s a bit shy and reserved with hearing loss would be, you know, you could get yourself in a lot of trouble.” (Participant 14)
“If I couldn’t hear [my healthcare providers], I guess I could say ‘excuse me, I didn’t hear what you said’, right? It’s kind of simple, I guess in a way . . . if they were talking to me and I don’t hear what they are saying, what’s the point of the communication? . . . I’m the one that is at the loss, right? As in, I don’t understand what they said, or I didn’t hear what they said and I need to kind of know because supposedly it’s my care.” (Participant 2)
“The nurse I’m dealing with has to know [about my hearing loss]. I tell them right up front . . . I take the initiative because this is for me. If I don’t have it, I die . . . you have to be aware of what you are being told all the time.” (Participant 3)
“[My nurses] know that I’m hard of hearing. I’ve told them all . . . I have no problem telling the nurses anything . . . I can’t help that my hearing is like that . . . I just tell them, ‘I’m a little hard of hearing, can you go out of your way to talk a little louder or something?’” (Participant 4)
“I tell them, you know. I’m not scared to say . . . I guess if a person comes here and he doesn’t know he’s got hearing loss, that might be a problem, but I know.” (Participant 5)
“I want to know what is going on, so I keep asking . . . if they are treating me, I want to know what they are saying. That’s it.” (Participant 8)
“I haven’t a clue [what an assistive listening device] is . . . it is about three times as loud as your voice . . . I would make use of that right away . . . that certainly would be [helpful].” (Participant 3)
“I’ve never seen [an assistive listening device] before . . . [it would be very helpful] . . . that would make it better for me.” (Participant 10)
“I didn’t even know there was [a captioning app] . . . absolutely [it would be helpful]. Do you think there is such an app?” (Participant 3)
“Maybe the staff should realize I have hearing aids.” (Participant 14)
“One time I told [the nurse] they had to charge [my hearing aid remote].” (Participant 11)
“I think when you are dealing with somebody like a healthcare worker who, by definition these days, is stressed to the max, you don’t want to make their job any harder, and so you are so concerned about that.” (Participant 13)
“Well, I have to imagine it gets annoying for them, and we all want to be friends with our nurses. That’s my intent. It’s got to be worthwhile for them to treat you . . . it’s always a good plan to be on good terms with your nurse and don’t get them angry.” (Participant 3)
“I don’t want to be a pain in the butt to anybody.” (Participant 4)
“I don’t want to put a burden when a girl has to speak into a speaker for me . . . I need to wear [my hearing aids]. It’s not fair to them . . . I know [the nurses] have got a job to do, so I don’t interfere unless I need them.” (Participant 5)
“The eyesight [issue] to me is more cumbersome because you just can’t see stuff and then you are really shut out, more so that the hearing [loss] . . . vision [loss] bums me. That’s the worst. It’s worse than the hearing [loss].” (Participant 10)
“I have a lot more problems with mobility than I do with hearing.” (Participant 13)
“Way back . . . I was really sick . . . this doctor would come in . . . I couldn’t hear anything he was saying . . . I was too sick to speak for myself, so I just let him say whatever he had to say and then he would leave. That was probably the only time that I can think of where I never said anything to him like ‘oh I’m sorry, I can’t hear you.’” (Participant 2)
“To me, the hearing [loss] is just a small thing compared to [my vision issues].” (Participant 7)
“I deal with [hearing loss]. I don’t think the hearing is going to get any worse, but the dialysis is killing me because it’s making me weaker all the time. And, then I have Parkinson’s and then I have bladder cancer. I think the hearing aids is one of my smaller problems.” (Participant 8)

### Communication Tools May Be Needed in Transitional or Clinically Complex Situations

Participants acknowledged that their communication needs were dynamic and tools may be helpful at certain timepoints. For example, when first starting hemodialysis or when their health status or treatment changed. “Originally at first, yes. [I had difficulty understanding]. Yeah, it would have been nice to have [help]” (Participant 1). One explained they recently had a medication changed and the risk of miscommunication. “If you don’t [hear] then you might get the wrong medication . . . then the blood work will probably go off the charts and I will be in danger of doing harm to myself.” (Participant 3)

Once patients become familiar with dialysis, and during stable periods, conversations were perceived as less complex. The need for communication tools similarly was reduced. “I can communicate with the nurses quite well. There’s not much dialogue between us . . . they have the same questions all the time” (Participant 4).

### Patients With Their Own Resources May Rely Less on Center-Provided Tools

Many of the participants used hearing aids and were less likely to perceive a need for communication tools on site compared to those without. “I’m pretty set with the hearing aids” (Participant 1). However, not all patients have hearing aids or can afford high-quality devices. “If I had two or three thousand dollars and I could afford [hearing aids], I would have bought them, but no way” (Participant 12). “When I started out with the cheaper version of hearing aids . . . background noise was a problem. With these, no, because I can tune it out” (Participant 1).

Similarly, some participants deferred communication to their support person, who regularly accompanied them to treatment. “My wife will answer the questions . . . [providers] will ask my wife instead of asking me. She is with me every day, every time we are in here, so she knows what is going on” (Participant 6).

### Awareness and Self-Advocacy for Support Varies Across Patients

Not all participants expressed the same level of concern with hearing loss and its impact on communication. Some patients passively accepted it (“I survive. It’s ok. I take it” (Participant 12)) while others were more assertive. “I tell [the nurses about my hearing loss] right up front. I take the initiative myself because this is for me” (Participant 3). Another noted, “I can see a person . . . that’s a bit shy and reserved, with hearing loss, would be . . . in a lot of trouble” (Participant 14).

A few participants were facing multiple illnesses and felt hearing loss was minor in comparison. *“*I don’t think the hearing is going to get any worse, but the dialysis is killing me . . . and, then I have Parkinsons . . . bladder cancer. I think the hearing [loss] is one of my smaller problems” (Participant 8).

Some participants felt that communication was solely the patients’ responsibility and didn’t want to ask for help. “I think a lot of it has to do with the person themselves to say . . . I didn’t hear you, can you repeat that . . . could you talk louder” (Participant 2). “I don’t want to [be] a burden when a [nurse] has to speak into a speaker for me . . . It’s not fair to them” (Participant 5).

Others hadn’t realized that communication tools were even a possibility and expressed enthusiasm at the thought. One participant was unable to purchase hearing aids but used an assistive listening device during the interview. “[The Pocketalker is] a godsend. I mean it, it’s a godsend. I can hear everything – it’s perfect. That’s all I need” (Participant 12). “You know, I didn’t even know there was [a captioning app] . . . that would be enormously helpful]” (Participant 3).

Beyond these main themes, some participants also noted that their communication experiences varied across providers. “Every [healthcare provider] talks a little quieter or a little louder. Some nurses are clear as a bell and some, you really have to really listen” (Participant 5). However, this observation was only briefly raised by a few participants and wasn’t linked to perspectives about communication tools. Thus, while it is important to recognize that communication experiences will also depend on provider characteristics, we didn’t include this as a key theme.

## Discussion

The objective of this qualitative study was to describe the communication-related experiences of patients with hearing loss when conversing with health care providers during hemodialysis treatment, focusing on perceptions about communication tools. Many participants were satisfied with communication and relied on strategies such as asking the provider to speak louder or move closer if needed. Perspectives about communication tools varied both within and across participants. Three themes were identified that describe these differences, as discussed below.

### Communication Tools May Be Needed in Transitional or Clinically Complex Situations

Hemodialysis is a complex treatment and patients receiving maintenance therapy experience a range of physical, emotional, and social impacts.^21^ When first starting treatment, patients often experience rapid health deterioration, changes in social roles, disruptions to daily life, and limited knowledge about disease management.^22^ Patients often report feeling overwhelmed^23,24^ and struggle to cope with their changing identity^24^ during this transition period. Cancer patients similarly experience fluctuations in their communication needs throughout their treatment journey.^25^ During the diagnostic and treatment initiation phases, patients’ communication needs are high but tend to reduce as they became less anxious and more informed.

Although hemodialysis treatment generally becomes habitual over time, patients often have multiple comorbidities, a high hospitalization burden,^26^ and complicated medication regimens.^27^ Therefore, even if a patient is not new to hemodialysis, conversations during treatment may become non-routine as health status evolves or medication changes are made. These heightened communication periods are not limited to those with hearing loss, however people with hearing loss are especially at risk of communication problems in health care.^28^ Therefore, ensuring that patients with hearing loss have the communication supports they need to properly engage during these situations is critical so that they can understand any new information, ask questions, and emotionally connect with their health care providers.

However, patients often develop strong relationships with their dialysis health care team members.^29^ Some may want to participate in conversations all the time due to the social benefits and not just during important clinical situations. Knowing individual patient preferences will help address this variation.

While the notion of privacy was not raised in our study, it is very likely that some patients would rather use a communication tool instead of asking a provider to speak louder. Patients with hearing loss have previously felt their privacy was compromised as health care staff raised their voices when speaking.^3^ Thus, offering tools for private conversations is similarly important.

#### Patients With Their Own Resources May Rely Less on Center-Provided Tools

Unsurprisingly, participants that used hearing aids were less interested in tools like assistive listening devices or captioning, compared to those without. A few participants commented that if hearing aids were more affordable, they would prefer those; however, something like a Pocketalker or captioning would be better than nothing.

Cost is a primary barrier to hearing aids. In Alberta, the cost of aids can range from CAD 1000 to 6000 and coverage varies considerably.^30^ Many people that require hearing aids are therefore unable to be treated. Although the most equitable solution is to remove financial barriers to hearing aid access, hemodialysis centers can nevertheless take meaningful steps to support patients who remain without them.

For example, real-time captioning has been used to improve how informed consent is obtained from patients with hearing loss prior to undergoing anesthesia.^31^ This tool may also be useful for other important conversations like discussing changes to medications, symptoms, or relationship-building. At minimum, providers can write out important information and make simple changes in body language or use speech strategies^32^ such as moving closer to the patient, speaking louder (depending on the type of hearing loss^33^) and repeating messages, removing masks (with the patient’s permission^34^), and taking more time to ensure information is understood.

Some hemodialysis patients often rely on a support person as a communication resource. In our study, three participants were accompanied by their spouse, who typically would communicate on their behalf. This is common among people with hearing loss and can lead to patients feeling dependent or lacking privacy.^35^ In addition, this reliance can affect support persons, as they often need to transform their lives to take on this role. “Third party disability”^36^ is a term that has been used to describe the impact that a partner’s hearing loss has on the unimpaired spouse’s lifestyle, communication, and emotional well-being. This may be especially present among people supporting hemodialysis patients, given the magnitude of caregiving required by support persons both in terms of time (three sessions per week, four hours per session) and responsibilities (e.g., dietary management, complication monitoring).^37^

Some patient-support person dyads may still prefer to attend hemodialysis together and collectively participate in discussions. However, if communication tools are provided to patients, support persons will no longer be relied upon. This can help patients feel more confident and independent and reduce the support person burden, benefiting both parties.^38^

### Awareness and Self-Advocacy for Support Varies Across Patients

The experience of hearing loss varies considerably across individuals, both in its severity^39^ and impact.^40^ Not surprisingly, participant perspectives about patient-provider communication and the need for support similarly varied. Some (mostly those with hearing aids) were satisfied and experienced little trouble. Others recognized they had communication difficulty but hadn’t considered asking for help either for fear of imposing or because they hadn’t been aware tools even existed.

Hemodialysis centers are often organized as shared, open-bay treatment areas where patients have direct visual access to nursing staff and rounding nephrologists. Participants in this study noted how busy the health care staff worked, resulting in them not asking for help. A study exploring patient perspectives of clinical communication in a major trauma center also found that when patients can observe staff members under pressure, they find it difficult to express their communication needs for fear of being a burden or nuisance.^41^

Some participants were more comfortable asking for help compared to others. Self-advocacy is shaped by multiple factors including stigma^42^ and knowledge of rights.^43^ While all participants in our study were comfortable disclosing their hearing loss to their providers, our recruitment approach may have missed patients that felt stigmatized and therefore kept their hearing loss private. We did, however, find that participants weren’t always aware that they could ask for help. Reminding patients about their right to hearing and understanding and letting them know what tools are available may help empower patients to receive better care.

Lastly, a few participants described feeling overwhelmed with competing health priorities and dismissed their hearing difficulties. Patients who receive outpatient hemodialysis care often experience high comorbidity and symptom burden^44,45^ and tend to prioritize symptoms with immediate implications for their physical status (e.g. fatigue, insomnia, mental health).^46,47^ As such, these patients may not seek help with hearing; however, may still have communication issues which could worsen their health and well-being. Ensuring that tools are readily available and easy for patients to use will reduce barriers for struggling patients.

#### Implications for Practice

The findings from this study offer important considerations for practice. Recommendations for health care providers working in outpatient hemodialysis centers are provided in Box 1.

**Box 1.** Recommendations: How Health Care Providers Can Support Patients With Hearing Loss in Outpatient Hemodialysis Centers.

1. Hearing loss isn’t always obvious, and it can be difficult to know when someone is having trouble hearing. Patients may not always disclose their hearing difficulties. Provider training on how to recognize patients with hearing loss can help identify and support patients who are less likely to advocate for their own communication needs. Providers should consider asking patients about their hearing needs when they first start treatment and document this in the medical record. 2. Hearing loss can range in severity and the level of support required will similarly be different. Some patients will have hearing aids and may not need communication tools. But there is great variation in the quality of hearing aids, so even if a patient is wearing a hearing aid, they may still have communication difficulties. Some patients will need hearing aids but are not able to afford them. Providers should not make assumptions about a patient’s hearing based on hearing aid usage or lack thereof. Providers should ideally have some familiarity with hearing aids so that they can help their patients to troubleshoot simple technical issues (e.g., spent battery) during treatment. 3. Some patients aren’t comfortable asking for help due to stigma, fear of imposing, or feeling overwhelmed with health issues. Providers and/or health care organizations should (1) reassure patients that being able to hear and understand properly is their right and will benefit them, (2) routinely offer easy to use tools and don’t wait for patients to ask, and (3) place visible signage throughout the center indicating that communication access is a right and that support is available (for example, displaying the international symbol for communication). 4. There are number of ways to help patients with hearing loss communicate. These range from speech strategies (e.g., moving closer, speaking slower, repeating) to tools (e.g., whiteboards, assistive listening devices, speech-to-text captioning). Patient preferences will vary. Most patients aren’t aware that communication tools are available. Health care sites should be equipped with an appropriate variety of such tools, whereas providers should inform patients about the different options and accommodate accordingly. 5. Patients’ communication needs can change from day-to-day and may heighten during critical situations. For example, when first starting hemodialysis or during changes in health or treatment. Hearing status can similarly fluctuate. Some patients will want to participate in convserations all the time, due to the social benefits and strong patient-health care provider relationship. Providers should routinely check in with patients to ask if they would like communication support, especially during critical periods. Visit the Alberta Health Services’ Communication Access website for more information and resources on how to support patients with hearing loss.

#### Limitations and Future Research

This study has some limitations that should be considered when interpreting the findings. First, we approached patients who were identified by nursing staff as having hearing loss. As a result, some eligible individuals may have been overlooked if their hearing loss wasn’t obvious to health care providers. The perspectives of these patients are important, as presumably also in care their providers don’t realize they have hearing loss, which would affect their communication experience and views on support. Second, while efforts were made to keep conversations private, it is possible that a relative lack of privacy in this setting may have influenced some responses. Third, no participants reported a language difficulty, used a hearing device other than a hearing aid (e.g., a cochlear implant), or identified as Deaf. People in these groups may have divergent perspectives, and thus the findings from our study may not be transferable.

Future research should explore the experiences of patients with hearing loss and language difficulties, those that use implantable devices (e.g., cochlear implants), patients belonging to the Deaf community, and support persons. Implementation studies that evaluate which communication tools are most helpful to support hemodialysis patients with hearing loss, and how they are best implemented, will be useful to inform practice change.

## Conclusions

Communication support needs are both person-specific and context-dependent, varying across and within patients. Communication tools may be useful in the outpatient hemodialysis context, depending on the clinical context, patients’ access to personal resources, and their awareness and self-advocacy. Not all patients that may benefit from communication support will request help. Hemodialysis centers should be equipped with a variety of tools and providers should routinely check in with each patient about whether support is needed, as their needs can vary.

## Supplemental Material

sj-pdf-1-cjk-10.1177_20543581261454470 – Supplemental material for Communication Experiences of Patients With Hearing Loss During Hemodialysis Treatment and the Potential Role of Communication Tools: A Qualitative Study Supplemental material, sj-pdf-1-cjk-10.1177_20543581261454470 for Communication Experiences of Patients With Hearing Loss During Hemodialysis Treatment and the Potential Role of Communication Tools: A Qualitative Study by Alex DeBusschere, Meaghan Lunney, Sonja Reid, Nancy Verdin, Shannan Love, Gillian Crysdale, Stephanie Thompson, David Nicholas, Tiffany Boulton, Kara Schick-Makaroff, Lorienne Jenstad, Sharon Straus, Jayna Holroyd-Leduc, Maoliosa Donald, Patti-Jo Sullivan, Tanis Howarth, Julie Evans and Marcello Tonelli in Canadian Journal of Kidney Health and Disease
