# Supplementary material for: Communication Experiences of Patients With Hearing Loss During Hemodialysis Treatment and the Potential Role of Communication Tools: A Qualitative Study
Source: Can J Kidney Health Dis. 2026 Jun 12;13:20543581261454470. doi: 10.1177/20543581261454470 (PMC13263498; doi:10.1177/20543581261454470)
Supplement: sj-pdf-1-cjk-10.1177_20543581261454470 – Supplemental material for Communication Experiences of Patients With Hearing Loss During Hemodialysis Treatment and the Potential Role of Communication Tools: A Qualitative Study [file sj-pdf-1-cjk-10.1177_20543581261454470.pdf]

Supplemental Material

Item 1. Interview guide

1. Tell me about your experiences with communication at the hemodialysis centre.

2. Do your healthcare providers here know that you have hearing difficulties?

3. Have your providers used any communication tools (for example, a microphone or writing things down)?

4. What makes communication harder for you while receiving hemodialysis?

5. Do you have to tell every new provider that you have hearing difficulties?

6. How do you think hearing loss affects your experience at the hemodialysis centre?

7. What would you change if you could?

8. What would you like your providers to know?

Table S1. Consolidated criteria for reporting qualitative studies (COREQ) checklist

Item No	Guide Questions/Description	Reported on Page #
**Domain 1: Research team and reflexivity**		
Personal Characteristics		
1. Interviewer/ facilitator	Which author/s conducted the interview or focus group?	4
2. Credentials	What were the researcher's credentials? E.g., PhD, MD	3
3. Occupation	What was their occupation at the time of the study?	3
4. Gender	Was the researcher male or female?	3
5. Experience and training	What experience or training did the researcher have?	3
Relationship with participants		
6. Relationship established	Was a relationship established prior to study commencement?	4
7. Participant knowledge of the interviewer	What did the participants know about the researcher? e.g. personal goals, reasons for doing the research?	4
8. Interviewer characteristics	What characteristics were reported about the interviewer/facilitator? e.g. Bias, assumptions, reasons and interests in the research topic	4
**Domain 2: study design**		
Theoretical framework		
9. Methodological orientation and Theory	What methodological orientation was stated to underpin the study? e.g. grounded theory, discourse analysis, ethnography, phenomenology, content analysis	3
Participant selection		
10. Sampling	How were participants selected? e.g., purposive, convenience, consecutive, snowball	4
11. Method of approach	How were participants approached? e.g., face-to-face, telephone, mail, email	4
12. Sample size	How many participants were in the study?	5
13. Non-participation Setting	How many people refused to participate or dropped out? Reasons?	5
14. Setting of data collection	Where was the data collected? e.g., home, clinic, workplace	4
15. Presence of nonparticipants	Was anyone else present besides the participants and researchers?	4
16. Description of sample	What are the important characteristics of the sample? e.g. demographic data, date	Table 1
Data collection		
17. Interview guide	Were questions, prompts, and guides provided by the authors? Was it pilot tested?	4<br>Suppl Item 1
18. Repeat interviews	Were repeat interviews carried out? If yes, how many?	4
19. Audio/visual recording	Did the research use audio or visual recording to collect the data?	4

Item No	Guide Questions/Description	Reported on Page #
20. Field notes	Were field notes made during and/or after the interview or focus group?	4
21. Duration	What was the duration of the interviews or focus group?	4
22. Data saturation	Was data saturation discussed?	4
23. Transcripts returned	Were transcripts returned to participants for comment and/or correction?	4
**Domain 3: analysis and findings**		
Data analysis		
24. Number of data coders	How many data coders coded the data?	5
25. Description of the coding tree	Did the authors provide a description of the coding tree?	5
26. Derivation of themes	Were themes identified in advance or derived from the data?	5
27. Software	What software, if applicable, was used to manage the data?	5
28. Participant checking	Did participants provide feedback on the findings?	5
Reporting		
29. Quotations presented	Were participant quotations presented to illustrate the themes/findings? Was each quotation identified? e.g., participant number	6-7, 21-24
30. Data and findings consistent	Was there consistency between the data presented and the findings?	6-7, 21-24
31. Clarity of major themes	Were major themes clearly presented in the findings?	6-7, 21-24
32. Clarity of minor themes	Is there a description of diverse cases or a discussion of minor themes?	7

From: Tong A, Sainsbury P, Craig J. Consolidated criteria for reporting qualitative research (COREQ): a 32-item checklist for interviews and focus groups. *International Journal for Quality in Health Care*. 2007. Volume 19, Number 6: pp. 349 – 357

Table S2. List of participants with demographics, personal communication supports, and years on dialysis

Participant ID	Age	Gender	Personal supports	Years on dialysis
1	51	Man	Hearing aids	4-6 years
2	79	Woman	None	1-3 years
3	72	Man	None	< 1 year
4	77	Man	Has hearing aids but doesn't wear them. Spouse usually attends treatment.	< 1 year
5	73	Man	Has hearing aids but doesn't wear them. Spouse sometimes attends treatment.	< 1 year
6	85	Man	Has hearing aids. Spouse usually attends treatment.	4-6 years
7	61	Man	Had hearing aids but lost them.	< 1 year
8	88	Woman	None	> 6 years
9	67	Woman	Has hearing aids but doesn't wear them.	1-3 years
10	76	Woman	Had hearing aids but lost them.	> 6 years
11	72	Man	Hearing aids	> 6 years
12	75	Man	Had hearing aids several years ago but not currently.	1-3 years
13	80	Man	Hearing aids	> 6 years
14	84	Woman	Hearing aids	1-3 years
